# Toll-Like Receptor 9 Promotes Cardiac Inflammation and Heart Failure during Polymicrobial Sepsis

**DOI:** 10.1155/2013/261049

**Published:** 2013-07-02

**Authors:** Ralph Lohner, Markus Schwederski, Carolin Narath, Johanna Klein, Georg D. Duerr, Alexandra Torno, Pascal Knuefermann, Andreas Hoeft, Georg Baumgarten, Rainer Meyer, Olaf Boehm

**Affiliations:** ^1^Institute for Physiology II, University of Bonn, Nussallee 11, 53115 Bonn, Germany; ^2^Department of Anaesthesiology and Intensive Care Medicine, University Hospital Bonn, Sigmund-Freud-Straße 25, 53127 Bonn, Germany; ^3^Department of Cardiothoracic Surgery, University Hospital Bonn, Sigmund-Freud-Straße 25, 53127 Bonn, Germany

## Abstract

*Background*. Aim was to elucidate the role of toll-like receptor 9 (TLR9) in cardiac inflammation and septic heart failure in a murine model of polymicrobial sepsis. *Methods*. Sepsis was induced via colon ascendens stent peritonitis (CASP) in C57BL/6 wild-type (WT) and TLR9-deficient (TLR9-D) mice. Bacterial load in the peritoneal cavity and cardiac expression of inflammatory mediators were determined at 6, 12, 18, 24, and 36 h. Eighteen hours after CASP cardiac function was monitored *in vivo*. Sarcomere length of isolated cardiomyocytes was measured at 0.5 to 10 Hz after incubation with heat-inactivated bacteria. *Results*. CASP led to continuous release of bacteria into the peritoneal cavity, an increase of cytokines, and differential regulation of receptors of innate immunity in the heart. Eighteen hours after CASP WT mice developed septic heart failure characterised by reduction of end-systolic pressure, stroke volume, cardiac output, and parameters of contractility. This coincided with reduced cardiomyocyte sarcomere shortening. TLR9 deficiency resulted in significant reduction of cardiac inflammation and a sustained heart function. This was consistent with reduced mortality in TLR9-D compared to WT mice. *Conclusions*. In polymicrobial sepsis TLR9 signalling is pivotal to cardiac inflammation and septic heart failure.

## 1. Introduction

Sepsis is still one of the major causes of death in noncardiac intensive care units worldwide [1, 2] due largely to development of multiple organ dysfunction syndrome (MODS) during the course of the disease. The most severe complication due to MODS is septic heart failure, which may aggravate hemodynamic instability and pose an independent risk factor for death [3].

While the innate immune response is a first line of host defence against invading pathogens, it also plays a prominent role in the development of sepsis. The mechanisms leading to heart failure and eventual death are still unclear, despite years of research. 

Today excellent evidence exists that toll-like receptors (TLRs) initiate an inflammatory response leading to clinical signs of sepsis and septic shock after binding bacterial degradation products such as lipopolysaccharide (LPS) or bacterial DNA [4, 5]. Earlier studies from our group have demonstrated *in vivo* and *in vitro* that these bacterial products are capable of inducing cardiac inflammation accompanied by significant reduction of cardiomyocyte contractility in different animal models [6–8]. These findings support the notion that somatic cells, such as cardiomyocytes, express TLRs and modulate the inflammatory response during sepsis in addition to immune cells. 

The complexity of clinical infections, which are often of polymicrobial origin, is not reflected when only one specific TLR agonist is applied. The gut is an especially common origin of polymicrobial infection [9]. A model to simulate peritonitis is colon ascendens stent peritonitis (CASP), which has been described as a reliable model of a progressing polymicrobial and generalized peritonitis with consecutive systemic sepsis [10]. We hypothesized that polymicrobial sepsis induces cardiovascular dysfunction via TLR9 and thereby causes septic heart failure. Thus, we investigated the effects of CASP surgery on cardiac inflammation and function in WT and TLR9-deficient (TLR9-D) mice.

## 2. Materials and Methods

### 2.1. Animal Handling and Care

Ten- to twelve-week-old C57BL/6 mice were purchased from Charles River (Bad Sulzfeld, Germany). TLR9-D mice were kindly provided by Professor Shizuo Akira (Osaka University, Japan) and back-crossed to a C57BL/6 background. Mice were housed in pathogen-free cages with free access to water and standard rodent chow ad libitum. This investigation conforms to the Guide for the Care and Use of Laboratory Animals published by the US National Institutes of Health (NIH publication number 85-23, revised 1996), and animal procedures were approved by the local committee for animal care (Landesamt für Umwelt, Natur und Verbraucherschutz, Recklinghausen, Germany).

### 2.2. CASP Surgery

To induce sepsis colon ascendens stent peritonitis (CASP) surgery was performed [10]. Animals were anaesthetised with 2 vol% isoflurane (Forene, Abbott GmbH, Ludwigshafen, Germany). After disinfecting the skin a small midline incision was made to open the abdominal wall. An 18-gauge stent was inserted into the ascending part of the colon approximately 1 cm aboral of the ileocaecal valve and fixed with 7/0 Ethilon thread (Ethicon, Norderstedt, Germany). To ensure safe intraluminal passage the stent was filled with a small amount of feces. The caecum was relocated into the abdominal cavity and the abdominal wall and skin were sutured with 5/0 Ethilon thread.

Sham surgery was carried out using the identical surgical procedures, but without stent implantation. Mice received a subcutaneous analgesic injection of 0.1 mg/kg bodyweight (BW) buprenorphine. Fluid resuscitation was administered by injecting 50 mL/kg BW of a 0.9% saline solution.

### 2.3. Bacterial Culture and Survival

At defined time points (6, 18, 24, and 36 h;  *n* = 15/group) after CASP the abdominal wall was incised again to flush the peritoneal cave with 3 mL of a sterile phosphate-buffered saline (PBS). This peritoneal lavage fluid was then aspirated into a sterile syringe. A serial log dilution was made, aliquots of which were then plated on Columbia blood agar with colistin and nalidixic acid (CNA) to detect Gram-positive bacteria and on MacConkey agar to detect Gram-negative bacteria. Both were incubated for 48 h at 37°C and the colony-forming units (CFUs) were then counted. 

A more detailed analysis of intestinal microbial flora was then performed for both groups (*n* = 6/group). The animals were anaesthetised with 2 vol% isoflurane and sacrificed. The colon was excised and 0.6 g of the feces was extracted from three different compartments of the intestine (ileum, caecum, and colon). Aliquots of these feces were diluted as described above and cultured on either MacConkey or Colombia CNA agar plates. After 48 h of incubation at 37°C the agar plates were inspected and bacterial growth was documented. The colonies were visually inspected and subcultured to allow the growth of bacterial monocultures. After further 24 h of incubation at 37°C biochemical analysis of the respective monocultures was performed (BD Phoenix 100, Becton, Dickinson and Company, Sparks, MD, USA).

In order to determine the impact of TLR9 on mortality after CASP WT and TLR9-D mice were monitored until 1 week after surgery in two additional experiments (*n* = 10/group).

### 2.4. Preparation of Hearts

Hearts were prepared immediately after sacrificing the animals. Beating hearts were transferred into cold PBS and allowed to beat spontaneously until no more blood was pumped out of the aortic root. They were then snap frozen in liquid nitrogen and stored at −80°C for further analysis.

### 2.5. mRNA and Protein Detection

Inflammatory parameters were detected via RT-qPCR in whole hearts of WT mice 6, 18, 24, and 36 h following CASP (*n* = 10/group). For RNA extraction the hearts were homogenized and RNA was isolated using the RNeasy Mini kit (Qiagen, Hilden, Germany). Ribonucleic acid was reversely transcribed using the High Capacity cDNA Reverse Transcription Kit (Applied Biosystems, Darmstadt, Germany; part number 4368814) according to the manufacturer's protocol. Real-Time qPCR was performed using TaqMan Gene expression Master Mix (Applied Biosystems, part number 4369016) with the following primers: CD14 (Mm00438094_g1), GAPDH (Mm99999915_g1), IL-1*β* (Mm99999061_g1), IL-6 (Mm01210732_g1), IL-10 (Mm00439616_m1), iNOS (Mm00440485_m1), TLR2 (Mm01213945_g1), TLR4 (Mm00445273_m1), TLR9 (Mm00446193_m1), TNF-*α* (Mm00443258_m1), and TREM1 (Mm01278455_m1). The reaction was processed in a TaqMan PCR system (Applied Biosystems), and the results were analysed based on the ratio between target accumulation and GAPDH accumulation.

ELISA was performed using Quantikine Mouse kits (R&D System, Abingdon, UK) for IL-1*β* (MLB00B) and IL-6 (M6000B) according to the manufacturer's protocol (*n* = 10/group).

### 2.6. Histology

Excised hearts were formalin-fixed (Anatech Ltd, Battle Creek, MI, USA) and embedded in paraffin [11]. Ten 5 *μ*m sections were mounted on glass slides at a 250 *μ*m interval. For MAC-2 staining sections below the papillary muscles were stained with MAC-2 clone 3/38 rat anti-mouse antibody for macrophages (Cedarlane, ON, Canada). As secondary antibody the Vectastain Elite ABC rat kit (rabbit anti-rat biotinylated IgG antibody) and diaminobenzidine (both from AXXORA, Lörrach, Germany) were utilized. Macrophage density was assessed by an investigator blinded to group assignment and expressed as cells/mm².

### 2.7. Recordings of Sarcomere Shortening

Sarcomere shortening of isolated ventricular cardiomyocytes from WT and TLR9-D mice was monitored in order to investigate whether bacterial components are able to impair cardiomyocyte function directly (*n* = 6–12/group). Murine cardiomyocytes were isolated as described elsewhere [12].

Cardiomyocytes of WT and TLR9-D mice were investigated immediately after isolation to determine baseline contractility. Treatment groups were incubated in Dulbecco's modified Eagle medium, supplemented with 5% minimal essential medium, 10% foetal calf serum, and 50 mg/mL gentamicin (culture media from Gibco, NY, USA) with and without heat inactivated bacteria for 4 h. The feces of the colon and caecum of 3 WT-mice was diluted in PBS and filtered through a 40 *μ*m mesh and finally heat inactivated at 80°C for two hours. Concentration of heat-inactivated bacteria (HIB) in the incubation solution was diluted until a 1 : 1 concentration of bacteria and cardiomyocytes was reached. This concentration was chosen according to preliminary experiments, in which the survival rate of cardiomyocytes after 4 h of incubation was tested.

Sarcomere shortening of ventricular myocytes was recorded using a video imaging system and SarcLen software as previously published (IonOptix, Milton, MA, USA) [8]. Regular striation patterns of the sarcomeres were analysed by fast Fourier transformation. The video system was mounted to an inverted microscope (Zeiss Axiovert 135TV, Jena, Germany, lens Fluar 40 1.3) and equipped with an experimental chamber containing permanent perfusion with Tyrode's solution (600 *μ*L/min leading to an exchange rate of three times per minute in the 200 *μ*L volume chamber) heated to 36°C. Independently of the pretreatment sarcomere shortening was continously monitored in Tyrode's solution (in mM: NaCl, 135; KCl, 4; CaCl_2_, 1.8; MgCl_2_, 1; HEPES, 2; glucose 10) to control for direct effects of the incubation medium on contractile response. Contractions were induced by bipolar external stimuli (0.4 ms, 30 V, SD9, Grass, Quincy, MA, USA). Stimuli were applied in pulse trains of 20 stimuli interrupted by 30 s stimulation pauses. The stimulation protocol for WT cardiomyocytes was 0.5, 10, 1, 8, 2, 6, and 4 Hz and for TLR9-D cardiomyocytes 0.5, 1, 2, and 4 Hz. In order to obtain representative shortening frequency relationships the five last shortenings of each train were averaged. The resulting signal was evaluated for the following parameters: resting sarcomere length, amplitude of sarcomere shortening, and maximal speed of sarcomere shortening and relengthening.

### 2.8. Hemodynamic Measurements

Measurements of cardiac function were performed with a 1.4 French pressure conductance catheter (SPR-839, Millar Instruments, Houston, TX, USA). Mice were anaesthetised with 2 vol% isoflurane. After preparation of the right carotid artery the catheter was inserted into the aortic arch and advanced through the aortic valve into the left ventricle. Pressure-volume signals were recorded under reduced isoflurane anaesthesia (1 vol%). During measurements body temperature was maintained at 36°C via a feedback loop consisting of a rectal temperature sensor coupled to a heating device. Pressure-volume signals were digitised using Powerlab (ADInstruments GmbH, Spechbach, Germany) and continuously recorded by Chart for Windows (Version 5.5.5 ADInstruments). Pressure-volume (P-V) loops were analysed with the PVAN 3.6 software package (Millar Instruments). The obtained conductance signal itself was not calibrated and contained parallel conductance signals from the surrounding tissue. Therefore, raw data were converted to absolute volume signals. Hence, we determined blood conductivity via a standard cylindrical calibration with fresh heparinised blood. In order to assess parallel conductance a 10 *μ*L bolus of hypertonic (10%) saline solution was injected into the left jugular vein at the end of the respective experiments. The following parameters were recorded: heart rate (HR), left ventricular systolic pressure (LVSP), stroke volume (SV), cardiac output (CO), maximum first derivative of systolic pressure rise with respect to time (d*P*/d*t*_max⁡_), maximum first derivative of diastolic pressure fall with respect to time (d*P*/d*t*_min⁡_), and stroke work (SW).

### 2.9. Statistical Analysis

Numerical results are given as mean ± standard error of the mean (SEM). For analysis of numerical data, Student's unpaired *t*-test was used to compare means between groups. In comparison with more than two groups 1-way ANOVA with Tukey's Multiple Comparison post hoc test was performed. Survival was plotted as a Kaplan-Meier curve and differences were analysed by log-rank test. Probability (*P*)  values <0.05 were considered to be statistically significant. Multiplicity adjusted *P* values were reported for a family-wise significance of 0.05 (95% confidence interval). Statistics were calculated using Prism 6.0 (GraphPad Software Inc., San Diego, CA, USA).

## 3. Results

### 3.1. Analysis of Bacteria and Animal Survival

We found no detectable differences in amounts of Gram-positive and Gram-negative bacteria between WT and TLR9-D mice (data not shown). Hence, TLR9 deficiency appears not to have a general impact on the commensal microbiotic flora in the intestine of mice.

An analysis of bacterial flora contained in the feces of each respective mouse genotype revealed a great number of Gram-negative bacteria containing Enterobacteriaceae in both mouse strains. In WT mice smaller numbers of *Proteus vulgaris, Staphylococcus sciuri, Enterococcus faecalis, Staphylococcus aureus, Enterococcus casseliflavus/gallinarum*, and* Lactobacillus* were found as well. In TLR9-D mice smaller numbers of *Staphylococcus simulans, Lactobacillus*, and* Enterococcus casseliflavus/gallinarum *were documented.

**Figure 1:**
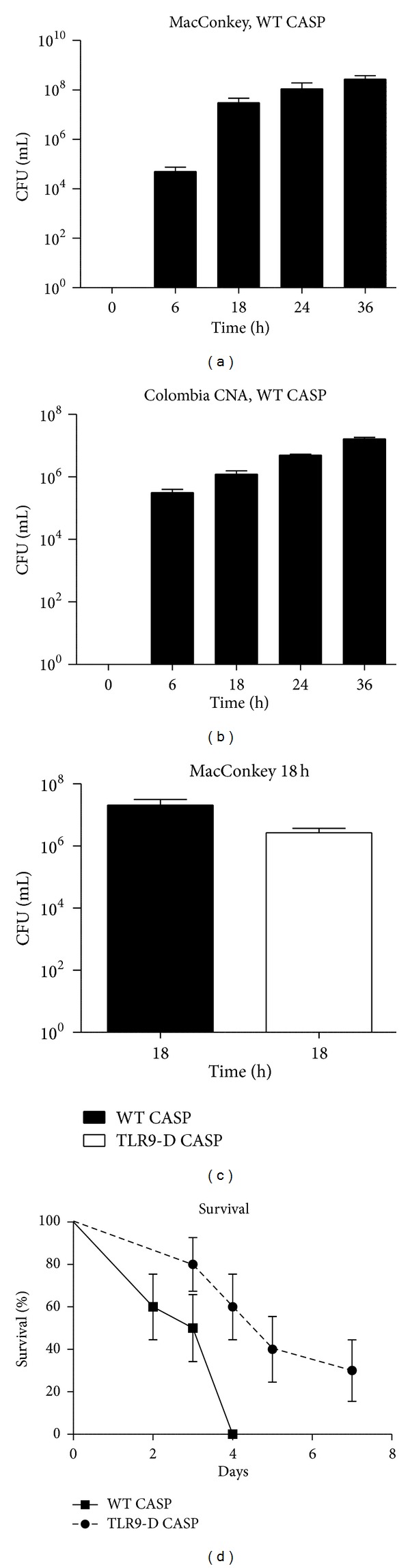
Intraperitoneal colony-forming units (CFUs) and survival after CASP. (a)  +  (b) Time course of CFUs on MacConkey (a) and Colombia + CNA (b) agar from peritoneal fluid of WT-CASP mice at 0, 6, 18, 24, and 36 hours after surgery (*n* = 15/group). (c) Comparison of intraperitoneal CFUs between WT CASP and TLR9-D CASP mice 18 h after surgery (*n* = 15/group). (d) Survival rate in WT CASP and TLR9-D CASP following surgery plotted as the Kaplan-Meier survival curve. Curves were significantly different (*P* = 0.0056;  *n* = 10/group; mean  ±  SEM).

Next, whether CASP induces a continuous release of bacteria into the peritoneal cavity was examined. We detected an increasing number of Gram-negative (MacConkey agar) and Gram-positive (Colombia+CNA agar) bacteria in the peritoneal cavity of WT mice from 0 to 36 h after CASP (Figures 1(a) and 1(b)). 

A comparison between WT and TLR9-D mice of intraperitoneal CFUs 18 h after CASP did not reveal any differences (*P* = 0.1089, Figure 1(c)). 

After CASP surgery WT animals showed continuously rising mortality with 100% of all mice being dead after 4 d. However, in TLR9-D mortality was significantly lowered (*P* = 0.0056) with 60% of the mice alive after 4 d and 30% of the TLR9-D animals survived 1 week (Figure 1(d)).

### 3.2. Cardiac-Specific Cytokine Profile after CASP in WT Mice

**Figure 2:**
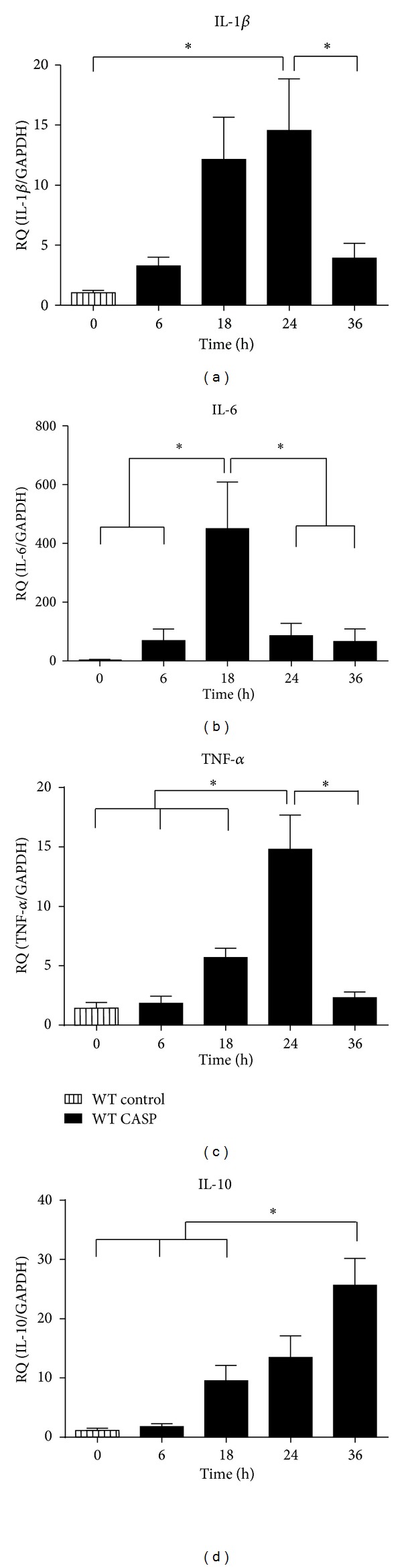
Cardiac inflammation profile. (a)–(d) Messenger RNA levels of pro- and anti-inflammatory cytokines obtained from WT mice hearts 6, 18, 24, and 36 hours after CASP surgery. Untreated WT mice served as control group (*:*P* < 0.05;  *n* = 10/group; mean  ±  SEM).

Inflammatory parameters were detected via RT-qPCR in whole hearts of WT mice 6, 18, 24, and 36 h following CASP. Interleukin- (IL-) 1*β* and tumor necrosis factor- (TNF-) *α* were upregulated in the hearts in a time-dependent manner within the first 24 h and declining to control levels in the following 12 h (IL-1*β* 24 h: versus control *P* = 0.0154, versus 36 h *P* = 0.0476, Figure 2(a); TNF-*α* 24 h: versus control *P* = 0.0011, versus 36 h *P* = 0.0007, Figure 2(c)). Interleukin-6 was significantly elevated only after 18 h (versus control *P* = 0.0091, Figure 2(b)). In contrast, anti-inflammatory cytokine IL-10 showed a continuous increase over time, finally reaching significance at 36 h *after infection *(versus control *P* = 0.0015, Figure 2(d)). Interestingly, iNOS mRNA expression was significantly upregulated in the heart only 6 h (versus control *P* < 0.0001, Figure 6(h)) after CASP surgery and returned to control level at the later time points. 

### 3.3. Immune Receptor Regulation in the Heart of WT Mice

**Figure 3:**
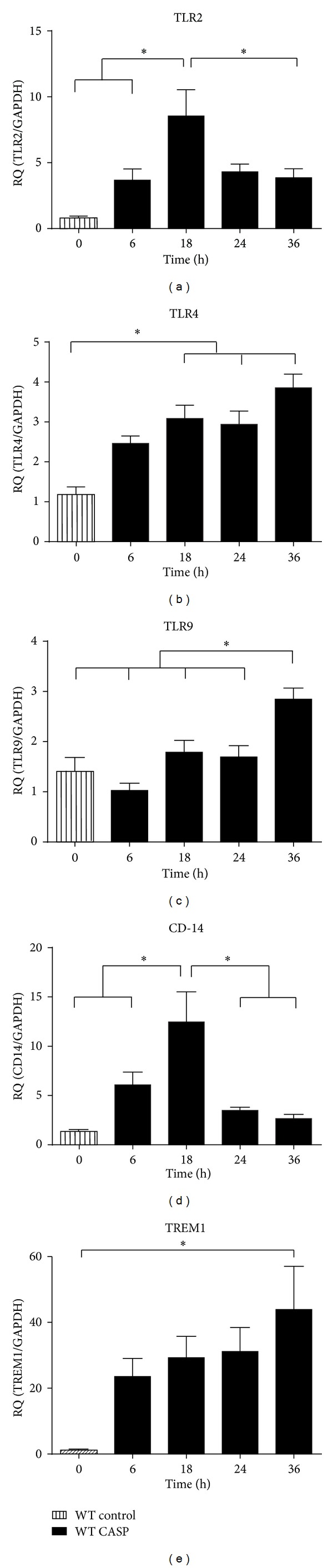
Immune receptor profile in WT hearts. (a)–(e) Messenger RNA levels of TLR2, 4, and 9, CD-14 (a)–(d), and TREM1 (e) were analysed 6, 18, 24, and 36 h after sepsis induction. Untreated WT mice served as control (a)–(e) (*:*P* < 0.05;  *n* = 10/group; mean  ±  SEM).

Eighteen hours after CASP TLR2 and CD14 were significantly upregulated (TLR2: versus control *P* = 0.0011, Figure 3(a); CD14: versus control 0.0003, Figure 3(d)). Toll-like receptor 4 was increased 18, 24, and 36 h after CASP (all versus control *P* = 0.0067, *P* = 0.0092, *P* = 0.0001, Figure 3(b)), whereas TLR9 was elevated only 36 h *after infection* (versus control *P* = 0.0029, Figure 3(c)). Triggering receptor expressed on myeloid cells 1 (TREM1) mRNA increased continuously reaching the level of significance after 36 h (versus control *P* = 0.0115, Figure 3(e)).

### 3.4. Cardiac-Specific Cytokine Profile 18 h after CASP in WT and TLR9-D Mice

Eighteen hours after sepsis induction WT mice displayed distinct clinical symptoms of systemic inflammation mirrored by increasing intraperitoneal bacterial load as described above. At this time point mortality was still moderate with about 20% WT animals being dead (Figure 1(d)). Therefore, this time point was chosen for a comparison of WT and TLR9-D animals. 

**Figure 4:**
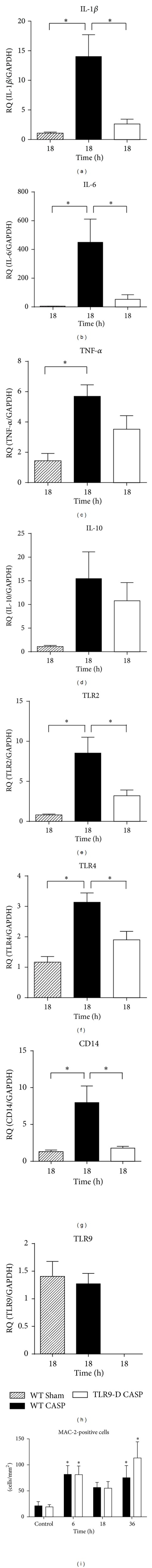
Comparison of inflammatory parameters in WT and TLR9-D mice. (a)–(h) Cardiac cytokine and TLR mRNA expressions were compared between WT Sham, WT CASP, and TLR9-D CASP hearts 18 h after surgery (*:*P* < 0.05; *n* = 10/group; mean  ±  SEM). (i) Quantification of MAC-2-positive macrophages in immunohistochemically stained sections of the heart of control and CASP mice 6 h, 18 h, and 36 h after surgery (WT: white bars; TLR9-D: black bars; *:*P* < 0.05 versus respective control; *n* = 6/group; mean  ±  SEM).

As demonstrated earlier, expression of pro- and anti-inflammatory cytokines as well as of PRRs was elevated in WT CASP mice 18 h after surgery compared to WT Sham (Figures 3(a)–3(e)). In TLR9-D CASP hearts all inflammatory mediators except for TNF-*α* and IL-10 (TNF-*α*: versus WT CASP *P* = 0.1295, Figure 4(c); IL-10 versus WT CASP *P* = 0.7286, Figure 4(d)) were expressed at a lower level than in WT CASP animals. Interestingly, TREM1 mRNA was significantly elevated in TLR9-D mice after CASP compared to WT Sham (*P* = 0.0463, data not shown). The expression of all other measured PRRs and cytokines remained unchanged in TLR9-D CASP hearts (all versus WT control: IL-1*β*: *P* = 0.8624, Figure 4(a); IL-6: *P* = 0.9431, Figure 4(b); TNF-*α*: *P* = 0.1689, Figure 4(c); IL-10: *P* = 0.3625, Figure 4(d); TLR2: *P* = 0.5343, Figure 4(e); TLR4: *P* = 0.2543, Figure 4(f); CD14: *P* = 0.9764, Figure 4(g)).

**Figure 5:**
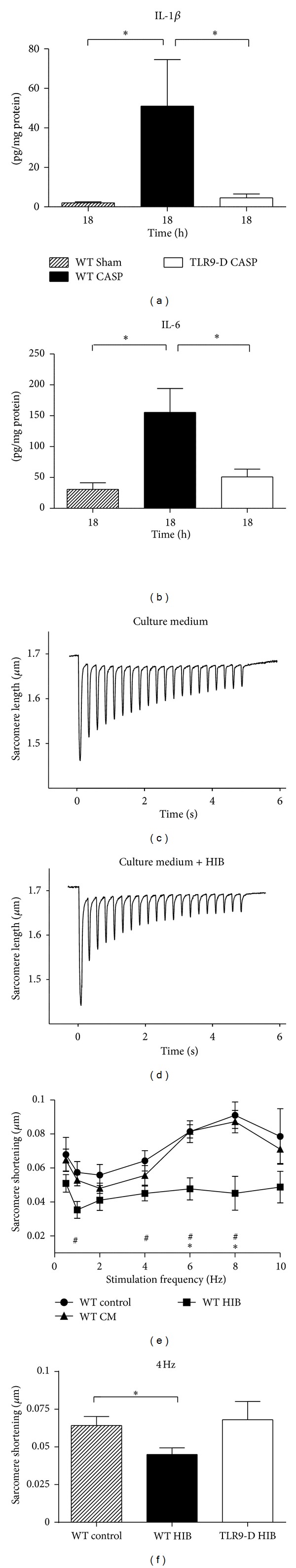
Protein levels of cytokines and cardiomyocyte contractility. (a), (b) Protein levels of proinflammatory cytokines were analysed via ELISA 18 h after CASP in hearts of WT Sham, WT CASP, and TLR9-D CASP mice (*:*P* < 0.05;  *n* = 8/group; mean  ±  SEM). (c), (d) Original recordings of sarcomere shortenings of isolated cardiomyocytes incubated in culture medium (CM) or culture medium + heat-inactivated bacteria (HIB) stimulated at 4 Hz. (e) Plot of averaged sarcomere-shortening amplitude versus stimulation frequency recorded directly after isolation (WT control) or after incubation with culture medium (WT CM) or with CM + HIB (^#^:*P* < 0.05 WT HIB versus WT control; *:*P* < 0.05 WT HIB versus WT CM;  *n* = 6–12/group; mean  ±  SEM). (f) Comparison of sarcomere-shortening amplitude at 4 Hz stimulation frequency of WT control cardiomyocytes and WT and TLR9-D cardiomyocytes incubated in HIB for 5–7 h  (*n* > 5; *:*P* < 0.05; mean  ±  SEM).

To further validate the mRNA results of proinflammatory cytokines ELISAs of IL-1*β* and IL-6 were performed 18 h after CASP. Like the mRNA expression also the protein levels of the investigated cytokines in WT animals were significantly elevated after CASP surgery in WT mice (both versus WT Sham: IL-1*β*: *P* = 0.0221, Figure 5(a); IL-6: *P* = 0.0083, Figure 5(b)). In TLR9-D CASP mice protein concentrations of both proinflammatory cytokines stayed at the level of WT Sham hearts and were significantly lower than in WT CASP hearts (both versus WT CASP: IL-1*β*: *P* = 0.0281, Figure 5(a); IL-6: *P* = 0.0024, Figure 5(b)).

### 3.5. Macrophage Infiltration

To investigate the influx of macrophages into the heart control sections and sections 0, 6, 18, and 36 h after CASP surgery were stained with antimacrophage antibody MAC-2. Macrophage densitometry revealed a significant increase of macrophage accumulation after 6 h in both WT- and TLR9-D animals compared to the respective control group (WT CASP: *P* = 0.0168; TLR9-D CASP: *P* = 0.0114, Figure 4(i)). 18 and 36 h after CASP macrophage density remained higher than in control mice reaching the level of significance only at 36 h (all versus respective control: WT CASP: 18 h *P* = 0.7948; 36 h *P* = 0.0089; TLR9-D CASP: 18 h *P* = 0.8124, 36 h *P* = 0.0083, Figure 4(i)). There were no differences in macrophage influx between WT and TLR9-D animals at any time point.

### 3.6. Sarcomere Shortening

The amplitude of sarcomere shortening depended on stimulation frequency. Following a stimulation pause a postrest shortening with elevated amplitude appeared. Subsequently, the occurrence of a negative or a positive staircase effect depended on the applied stimulation frequencies (negative staircase:  <6 Hz; positive staircase:  >6 Hz; Figures 5(c) and 5(d)). 

Incubation of cardiomyocytes with heat-inactivated bacteria depressed steady-state shortening (Figures 5(c) and 5(d); culture medium 0.5 *μ*m, HIB 0.3 *μ*m at 4 Hz). However, neither frequency-dependent shortening behaviour nor resting sarcomere length was influenced by the polymicrobial stimulus.

In plots of steady-state shortening amplitude versus stimulation frequency shortening amplitude exhibited a biphasic pattern with a negative shortening-frequency relationship under 2 Hz and a positive relationship above 2 Hz. At 2 Hz shortening was minimal (Figure 5(e)). This pattern was comparable in cells measured directly after isolation and those preincubated in culture medium for 4 h (Figures 5(c) and 5(d)). The addition of bacteria caused a decrease of shortening amplitude at all frequencies being significant at 1 (versus control *P* = 0.0152), 4 (versus control *P* = 0.0335), 6 (versus control *P* = 0.0025), and 8 Hz (versus control *P* = 0.0073) (Figure 5(e)). As the relative depression of sarcomere shortening was high at frequencies above 4 Hz the frequency dependence of shortening was abolished. Cardiomyocyte shortening of TLR9-D cells was also recorded after incubation in culture medium and culture medium supplemented with HIB at stimulation frequencies between 0.5 and 4 Hz. In contrast to WT cardiomyocytes TLR9-D cells proved to be insensitive to HIB incubation (WT HIB versus WT control *P* = 0.0477, TLR9-D HIB versus WT control *P* = 0.9334; Figure 5(f)). 

The speed of sarcomere shortening as well as relengthening showed the same frequency relationship as the sarcomere-shortening amplitude. Bacterial incubation suppressed both parameters significantly (data not shown).

### 3.7. Hemodynamic Measurements

**Figure 6:**
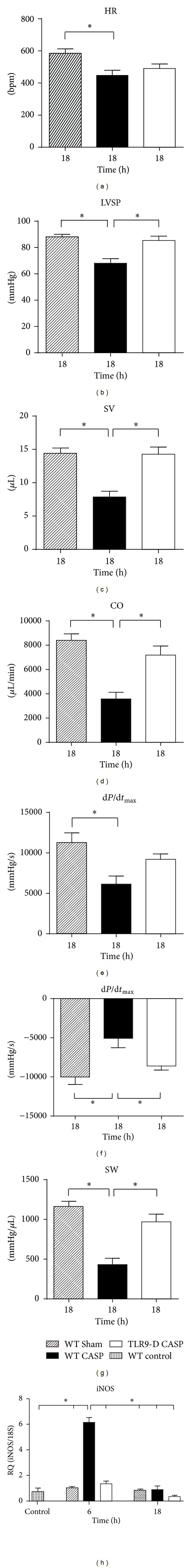
Left ventricular recordings of hemodynamic parameters and RT-qPCR of iNOS expression 18 hours after CASP surgery in the heart. (a)–(g) The measured parameters were (a) heart rate (HR), (b) left ventricular systolic pressure (LVSP), (c) stroke volume (SV), (d) cardiac output (CO), (e) maximal first derivative of pressure rise (d*P*/d*t*_max⁡_), (f) maximal first derivative of pressure fall (d*P*/d*t*_min⁡_), and (g) stroke work (SW). (h) Time course of iNOS mRNA expression in the heart in WT and TLR9-D mice 6 and 18 h after CASP. (*:*P* < 0.05  *n* = 8/group; mean  ±  SEM).

Hemodynamic parameters were recorded *in vivo* by means of a pressure-volume catheter. CASP-induced inflammation depressed all monitored hemodynamic parameters in WT mice 18 h after surgery (all versus WT Sham HR: *P* = 0.0177; LVSP: *P* = 0.0009; SV: *P* = 0.0009; CO: *P* = 0.0004; d*P*/d*t*_max⁡_:  *P* = 0.0066; d*P*/d*t*_min⁡_:  *P* = 0.0056; SW: *P* = 0.0001; Figures 6(a)–6(g)). This sepsis-dependent cardiac depression was less pronounced in TLR9-D animals and never reached significance compared to WT Sham. On the contrary, LVSP, SV, CO, d*P*/d*t*_min⁡_, and SW were significantly better in TLR9-D mice than in the WT CASP animals (LVSP: *P* = 0.0016; SV: *P* = 0.0004; CO: *P* = 0.0028; d*P*/d*t*_min⁡_:  *P* = 0.0347; SW: *P* = 0.0010). Interestingly, iNOS mRNA expression increased significantly in WT CASP hearts only 6 h after surgery (versus WT control *P* < 0.0001, Figure 6(h)), returning to baseline levels after 18 h (versus WT control *P* = 0.9985, Figure 6(h)).

## 4. Discussion

The main result of this study is that in the situation of a steadily increasing bacterial load in the peritoneal cavity TLR9 signalling is the main determinant for cardiac inflammation and consecutive cardiac dysfunction. Furthermore, the involved PAMPs interact directly with TLR9 on the cardiomyocytes. These findings complement and continue earlier work of our group demonstrating that specific TLR9 stimulation is able to induce cardiac inflammation *in vitro* and *in vivo* and to impair cardiac function *in situ* as well as sarcomere shortening of isolated cardiomyocytes. Cardiac depression could be antagonised by specific iNOS inhibition *in vitro* and TLR9 antagonism *in vivo *[7, 13]. In addition, we have shown that TLR9 stimulation is pivotal for vascular dysfunction during sepsis [14]. Interpretation of the results of the present study hinges on the composition of the sepsis-inducing stimulus. Therefore, we characterised the intestinal microbial flora of both genotypes. The intestines of both mouse strains were mainly colonized by Gram-negative Enterobacteriaceae. Although there were some differences in bacterial species, the overall picture was comparable. To resemble polymicrobial sepsis originating from the peritoneal cavity it is imperative to ensure an increasing amount of bacterial density [15]. This was confirmed by the increasing number of CFUs in bacterial cultures of peritoneal lavage. The number of CFUs did not differ significantly across genotypes at the investigated time point 18 h after surgery. Hence, neither the composition nor the extent of the inflammatory stimulus varied greatly between WT and TLR9-D mice.

In a murine model of polymicrobial sepsis induced by cecal ligation and puncture (CLP) Plitas et al. recently described lower bacterial counts in the peritoneal cavity of septic mice deficient in TLR9 [16], which contrasts with our findings. This might be explained by distinct differences in the surgical models used: CLP often induces a mere abscess formation without a continuous increase in CFUs over time [10]. Small differences in bacterial clearance in the peritoneal cavity may be detected more easily in the case of CLP-dependent short-term bacterial release compared to the case of cumulative bacterial load as caused by CASP. This short-term bacterial release is mirrored by an early upregulation of cytokines during CLP and has already been demonstrated by Williams et al. [17]. Therefore, a relatively early time point for the measurement of functional parameters in models of CLP (6 h) was chosen by different investigators [18, 19]. 

In our study survival of WT animals decreased continuously with all mice being dead 4 days after induction of polymicrobial sepsis. However, TLR9-D mice exhibited better survival with about 20% animals still alive after 1 week. This is in contrast to survival after CLP-induced polymicrobial sepsis, in which all WT animals died within 50 h. Here, 75% of TLR9-D mice survived this time span with no further cases of mortality [16]. This might again be explained by an excessive single stimulus after CLP compared to a slow increase of bacterial load after CASP. Interestingly, the overall mortality in control animals in our lab was higher than that in the report of Maier et al. [10]. This may be attributed to different housing conditions, for example, low-germ environment in our animal facility compared to a conventional breeding facility as mentioned by Maier et al. Due to a variable germ load in the housing facility the immune system of the mice may be able to handle higher bacterial loads more or less efficiently. Additionally, the virulence of the liberated bacteria from the gut may depend on the feeding conditions. The basic immunological finding of increased survival of TLR9-D mice during polymicrobial sepsis concurs in both models. However, the focus of the present study was septic heart failure in polymicrobial sepsis and the implication of TLR9 in the progression of this disease. 

In WT mice we detected an induction of proinflammatory cytokines such as TNF-*α*, IL-1*β*, and IL-6 within the first 24 h after CASP. This cytokine profile corresponded with the decline of cardiac function in WT animals 18 h after surgery. Increased levels of both IL-1*β*, and TNF-*α* have been associated with a depression of cardiac function [20]. Furthermore, IL-6 has also been linked to cardiac dysfunction during sepsis [21]. Thirty-six hours after sepsis there was a return to baseline of all proinflammatory parameters and an increase of the anti-inflammatory IL-10. Interleukin-10 has been demonstrated to be cardioprotective in several experimental settings, such as ischemia/reperfusion [22] and models of hypertrophy [23]. It has been described to improve left ventricular function by suppression of NF*κ*B activation, thereby interrupting TLR-dependent inflammatory signaling [24, 25].

It has been reported that TLR2 and TLR4 are regulated in the lung, liver, and spleen in mice suffering from polymicrobial sepsis [26]. Here, permanent upregulation of TLR4 and transient upregulation of TLR2 and CD14 mRNA expression in the heart peaking 18 h after CASP surgery were demonstrated. Both proteins have been found to be regulated in other models of TLR stimulation as well [27]. Recently, CD14 has been shown to be of pivotal importance not only for TLR4-dependent signalling, but also for the inflammatory response via TLR9 and TLR7 [28]. Toll-like receptor 9 was regulated primarily after 36 h. 

There is increasing evidence that TREM plays an additional role in the host defense against microbial invasion [29]. In our study cardiac TREM1, which is an amplifier of the systemic inflammatory response syndrome, continuously increased over time in WT mice after CASP surgery reaching the level of significance after 36 h. Toll-like receptor 9 deficient animals also showed a significantly elevated TREM1 mRNA expression 18 h after CASP surgery. However, TREM1 mRNA expression in TLR9-D was lower than that in WT hearts (data not shown). 

Macrophage infiltration into the heart taken as a sign for cardiac inflammation was induced in both WT and TLR9-D mice by polymicrobial sepsis. As significant differences between WT and TLR9-D hearts could not be detected in macrophage infiltration these cells do not seem to be an important source for the secretion of cytokines in our setting. 

For an understanding of reduced cardiac function it is important to know whether the cardiomyocyte itself is directly influenced by bacterial components or indirectly by inflammatory mediators originating from immune cells. Therefore, sarcomere shortening of isolated cardiomyocytes as well as intracardiac functional parameters were recorded. Here, we show that bacterial components depress sarcomere shortening of isolated cardiomyocytes. This can be taken as a sign of direct interaction between pathogen-associated molecular patterns (PAMPs) and the cardiomyocyte. Interestingly, HIB incubation of TLR9-D cells did not influence sarcomere shortening. This finding extends earlier results from our group that monovirulent stimuli (LPS and CpG-ODN) depress cardiomyocyte function via TLR4 [8] or TLR9 [7] on cardiomyocytes. Thus, it may be expected that both direct interaction between the polymicrobial stimulus and the cardiomyocyte itself and increased cytokine levels originating from immune cells can contribute to septic heart failure. 

Strikingly, cardiac function of WT mice with polymicrobial infection deteriorated in all recorded parameters compared to WT Sham animals and infected TLR9-D mice. This was in agreement with the expression profile of proinflammatory cytokines. Measures of *in vivo* cardiac contractility, d*P*/d*t*_max⁡_ and d*P*/d*t*_min⁡_, were reduced in polymicrobial sepsis. This may be attributed to the impaired cardiomyocyte function described above. The observed reduced left ventricular systolic pressure depends on two parameters, that is, cardiac contractility as well as vascular tone. Recently, it has been shown that polymicrobial sepsis leads to lower vascular contractility [14], which in turn depends mainly on TLR9 signalling. Interestingly, it was possible to antagonise vascular inflammation as well as depression of vascular contractility by applying a specific TLR9 antagonist, H154-thioate. Furthermore, H154-thioate was also able to antagonise cardiac depression caused by a specific TLR9 stimulation *in vivo* [13]. In addition to the direct antagonism of TLR9 pharmacological priming with TLR9 agonists prior to CLP has been demonstrated to prevent cardiac dysfunction by Gao et al. [18]. Pharmacological priming induces the PI3K/Akt pathway thereby attenuating myocardial apoptosis and thus preserving cardiac function. 

In addition to myocardial apoptosis an increased concentration of nitric oxide can impair cardiac contractility through activation of protein kinase G and by Ca^2+^ desensitization of Troponin [30, 31]. These findings were corroborated by the finding that LPS and CpG-ODN-induced impairment of cardiomyocyte sarcomere shortening are antagonised by the blockage of iNOS with S-methyl-isothiourea (SMT) *in vitro* [7, 8]. A significant early but transient upregulation of iNOS mRNA after 6 h in the hearts of septic WT mice indicates an iNOS-dependent mechanism contributing to cardiac dysfunction in our model of polymicrobial sepsis. However, the iNOS protein expression should last longer than the respective mRNA expression and may thus at least in part explain the cardiac depression measured 18 h after CASP. In addition, polymicrobial sepsis may induce further processes like adrenomedullin [32] or kynurenine expression [33], which by themselves downregulate systemic blood pressure.

## 5. Conclusions

Taken together TLR9 signalling seems to be of critical importance in polymicrobial sepsis as its absence increases survival of the animals dramatically via reduced cardiac inflammation. This attenuated cardiac inflammatory response allows the organism to preserve cardiovascular function, ultimately leading to better survival. Thus, pharmacological intervention of blocking TLR9 signalling shows promise as a new approach for treating polymicrobial sepsis.
